# A Practical Treatise on the Diseases Peculiar to Women, Illustrated by Cases Derived from Hospital and Private Practice

**Published:** 1845-04

**Authors:** 


### Art. II.

*A Practical Treatise on the Diseases peculiar to Women, illustrated by Cases derived from Hospital and Private Practice.* By Samuel Ashwell, m.d. Member of the Royal College of Physicians, Obstetric Physician and Lecturer to Guy's Hospital.—*Lond.* 1844. 8vo, pp. 754.

During the somewhat tardy publication of the successive parts of this work we have noticed its progress, and briefly mentioned the topics on which it treats. Now, however, we are enabled by the completion of the treatise to form an opinion of it as a whole, and to judge how far Dr. Ashwell has succeeded in accomplishing the purpose with which he commenced it some years ago.

"My aim," he says, in the preface to Part I, "has been to produce a treatise on female diseases, so true, simple, and practical, that it may form a safe and efficient guide to the elucidation and curative treatment of many at least of these intricate, rapidly progressing, and dangerous maladies."

Such was his purpose; it is no more than justice to say that he has fully performed it. The book is, what its author strove to make it, essentially a practical treatise; a simple, truthful record of the results of twenty years' experience. To the young practitioner it will be invaluable, since it supplies for the first time a want which he must long have felt, while those who have had most experience will yet find something to learn, much to commend in a book which shows so much patient observation, practical skill, and sound sense. But our praises may seem to require some justification; the best we can offer will be given by as complete an analysis of the work, as our limits will allow.

The present volume treats almost exclusively of the diseases of the unimpregnated state; those incidental to pregnancy and childbed are announced as the subject of a second. Dr. Ashwell has not, however, confined himself quite strictly to this arrangement, having introduced a notice of one or two morbid conditions which derive their chief importance from being complicated with pregnancy. The classification of diseases of the uterine system adopted by the author, is the simple division into functional and organic. The functional are mainly dependent on derangements of menstruation, which Dr. Ashwell regards as of more importance when they affect the regularity of its return, than when they merely alter the quantity or quality of the secretion. We cannot avoid noticing how this fact tends to substantiate Bischoff's and Raciborski's opinions with reference to the cause and signification of the menstrual flux. It is to be supposed that when the constitution is so far disordered as to interrupt the regularity of the periodical maturation and discharge of ova, there must be some graver cause in operation than would suffice to modify the character or quantity of the sanguineous discharge with which this process is attended.

The first functional disease treated of is *chlorosis*, which is distinguished into the mild, the inveterate or confirmed, and the complicated variety. The complication of chlorosis with amenorrhea is one of the most common forms, and by no means the least dangerous, since it not unfrequently happens that when long continued it gives rise to disease of some important organ, as the brain ; or of the digestive apparatus, or of the lungs, and thus induces a fatal issue. Its association with hematemesis is not unusual where amenorrhea has existed for some time ; but though patients are often reduced by the hemorrhage to an alarming state of anemia, this is a form of disease which under judicious treatment generally has a favorable termination. It is, however, a form often mismanaged, and that not merely by the inexperienced practitioner. The remarks of Dr. Ashwell on this very point, which follow the relation of some cases, are so much to the purpose that we subjoin them here :

'Hematemesis occurs more frequently than is supposed; and in connexion witn so much pain, fulness, and congestion in several organs as might appear to justify active treatment. I have seen bleeding, purging, and lead, lavishly employed; but with decidedly bad effect. In all the four cases narrated, there was anemia, quick, irritable pulse, and excitement, precisely the symptoms of chlorosis, and such as may without difficulty be distinguished from similar symptoms dependent on acute inflammatory disease. The transient neuralgic character of the chlorotic pains, notwithstanding their severity, the amenorrhea, countenance and pulse, must lead to a correct diagnosis, and to modified and local treatment. The great indication is, either to establish or to restore the catamenial function, and to attempt the attainment of this point, even by the empirical use of emmenagogues, bad as the practice may be, is less injurious than a full pursuance of the antiphlogistic plan. Bloodletting can seldom be required. On one occasion I visited a chlorotic patient who had been bled from the arm for the relief of thoracic fulness and difficult respiration ; she was partially and temporarily relieved. It was thought advisable to repeat the bleeding; and nothing could be more conspicuous than its bad effects. Her prostration of strength was extreme; the breathing was more laborious; and an anasarcous state of the body was universally apparent. Nor is it less important to reiterate the caution against excessive purging, especially where mercurial or drastic medicines are employed. The first object, doubtless, is to procure, by proper aperients, healthy and regular evacuations; but the anemia of the patient must be increased by their undue exhibition—a practice so common that some individuals doubt whether more harm than benefit has not accrued from their use. Let this be as it may, it is quite true, that the evil results of such a plan are not confined to the stomach and bowels themselves; the irritation and flatulent distention of the intestines leading to aggravation of the chlorosis, and to nervousness and distressing sinking, very difficult to be borne; and yet with such an increase of disorders, I have known mercury and aloes persevered in for weeks. So strong is the prejudice in favour of a 'good active purgation.'" (pp. 42-3.)

The other forms which he notices are chlorosis with chronic derangement of the digestive organs, with functional affection of the cerebrum, which sometimes reaches a most distressing severity, and continues for months or even years, without giving rise to structural change, with ascites, and with disease of the lungs. These complications do not occur indifferently at all ages, but some are more frequent at one time of life, others at a different period. Chlorosis alone, independently of amenorrhea, is a disease of early life. Associated with suppression of the menses it may be met with at all ages; but is seldom found conjoined with phthisis, except between puberty and the thirtieth year. The author's general observations on the treatment of chlorosis are such as might be expected from an experienced physician. His recommendation of the iodide of iron in cases of chlorosis associated with a strumous habit may be new to some of our readers. Dr. Ashwell employs it in the following form:

℞ Ferri Iodidi, gr. xvj.
Tinct. Calumbæ vel Gent. Co. ℥j.
Aquæ Destillatæ, ℥vij. ft. Mist.
Sumat cochl. ij magna bis terve quotidie.

The chapter on *amenorrhea* contains a careful examination of the various remedies that have been reputed to possess emmenagogue properties, which the young practitioner may often consult with advantage. Under the head of *vicarious menstruation*, the author mentions the occurrence of leucorrhea in place of the natural uterine secretion. He most frequently met with it in delicate and susceptible girls at the commencement of menstruation, or in weak and exhausted women. It occurs at the regular period, and may last as long, and be as abundant as the natural secretion, though quite devoid of colour. In cases were menstruation does not appear, the periodical return of this discharge removes all suspicion of congenital defect or malformation. The constitution too does not suffer as in chlorosis and amenorrhea, provided there be no excessive mucous discharge in the intervals of this periodical secretion. It is seldom, however, unless change of air be tried, or some appropriate course of treatment adopted, that it is succeeded by the flow of the catamenia, though to a certain extent the views of Dewees may be correct, who regarded it as a slow development of the menstrual function. Its occurrence is associated with general debility, and requires the use of tonics, especially iron, and of other means calculated to impart constitutional vigour, and thus to induce healthy menstruation.

The chapter on *dysmenorrhea* is followed by a collection of formulæ, and a hundred pages further on, we meet with a second; both very useful, but out of their proper place, which should be either at the beginning or end of the volume.

From *menorrhagia*, the author passes by a natural transition to the subject of *leucorrhea*; in which disease the mucus furnished by the lining membrane of the uterus, vagina, or other parts of the generative apparatus is increased and altered. He divides it into the *common* form, which is often *mild*, though sometimes *acute*, the *inveterate* and *chronic*, and the *symptomatic* leucorrhea; rejecting the arrangement into the uterine and vaginal varieties. The acknowledged difficulty of ascertaining, in a large number of cases, from which of these two sources the discharge flows, is his reason, and we think a valid one, for preferring a classification founded on the severity of the symptoms, and the consequent difference in the treatment required. Leucorrhea indeed may continue for a very considerable time, and be attended with symptoms of great severity, without giving rise to any change of structure. The supposed frequency of affection of the os and cervix uteri in connexion with leucorrheal discharges, which has been regarded as furnishing one of the most valid reasons for a far more general use of the speculum than is accordant with English feelings, is regarded by Dr. Ashwell as a mistaken notion. The cervix uteri indeed is occasionally soft, and the os patulous, or, at least, these tissues are all found to be relaxed. In several instances in which he used the speculum, the cervix was pale; in more acute cases slightly red; twice he found it of a deep crimson hue; but he never observed either erosion or ulceration, except when there was suspicion of venereal taint.

Besides the more ordinary forms of leucorrhea, there is one variety which Dr. Ashwell considers to be somewhat allied to hydrometra, "inasmuch as the contents of the uterus in the latter disease are not always serous, but sometimes albuminous or muco-purulent secretions." The description he gives of it, however, tallies exactly with Sir C. Clarke's account of "inflammation of the mucous membrane of the uterus terminating in secretion of pus."* Neither do we see any reason for rejecting Sir C. Clarke's supposition as to the nature of the affection; and to the mode in which the matter is prevented escaping by contraction of the cervix, or by adhesion of its walls as the result of inflammation, or by the mucus of the glands of the cervix obliterating its aperture.

*Inflammation of the cervix uteri*, so ably described by Sir C. Clarke, is conceived by Dr. Ashwell to be of very unusual occurrence. He states that "out of nearly 1000 cases of sexual disease, treated at Guy's, I find that inflammation of the os and cervix has happened only twenty times." This fact is important, for many practitioners we believe form an exaggerated estimate of the frequency of its occurrence.

A short chapter is devoted to the diseases attendant on the *decline of menstruation*; in which we find some very wise cautions with reference to their management. The writer points out the serious mischief that may result from attributing them without the most accurate enquiry to debility, rather than to repletion.

> "Let it be remembered that an accustomed evacuation is about to cease, or has finally disappeared; that the patients have been previously healthy, and that the probability therefore is that the weakness is apparent not real. If, for instance, because there is langour and inactivity, a slow pulse, torpid bowels, and depression of mind, stimulants and generous diet are allowed, some important organ will become congested, the brain or the lungs, and either suddenly fatal or structural disease may occur. I know not how often, but certainly very frequently, such errors happen; and it is therefore the more necessary to urge especial caution." (p. 200.)

He next lays down clearly, but in a few words, the golden mean to be observed in their treatment; and then proceeds to give an account of *hysteria*, concerning which affection he says well all that on a subject so trite there remained to say.

* On Diseases of Females, Part ii, p. 153.

That distressing complaint, the *irritable uterus*, to which the late Dr. Gooch first directed attention, comes last on the author's list of functional diseases. But though he classes it, in accordance with common opinion, among functional disorders, he does not seem inclined to subscribe to the notion of its purely neuralgic character, but is disposed, in common with Dr. Dewees and Dr. Lee, to regard it as a modified form of inflammation of the cervix uteri. He rests his opinion as well on the symptoms of redness, heat, permanent pain and tenderness of the cervix, which attend the malady, as on the nature of the remedies,—cupping, bleeding, aperients, and spare diet, which, even in Dr. Gooch's own cases, appear to have been of essential service. When long continued, indeed, he believes that it induces organic changes, and relates at p. 244, an instance in which induration and ulceration of the cervix supervened after six years of suffering from hysteralgia. A slight degree of prolapsus uteri is often associated with it, and Dr. Ashwell has often found that the introduction of a pessary has afforded great relief, not only in cases when this existed, but even when there was no marked uterine descent. Local depletion is often very serviceable, and within the last few years he has made frequent use of scarifications of the os uteri, which he practices in the manner recommended by the late Mr. Fenner. A suitable speculum is introduced into the vagina, and seven or eight crucial incisions are made with a cataract knife, and will frequently yield three or four ounces of blood. The operation is painless, and the wounds speedily cicatrize, and Dr. Ashwell states that he has found this mode of depletion prove most useful, not only in immediately relieving the characteristic pain, but also in restoring the uterus and especially its cervix to a healthy state.

The subject of *organic diseases of the uterus* is introduced by some very sound and practically useful remarks on their symptoms, diagnosis, pathology, and prognosis; and on the means of investigating uterine disease Some of our readers will probably feel surprise at finding hard tumours of the walls of the uterus,—the fleshy tubercle of many writers,—classed by Dr. Ashwell among malignant diseases. High authorities are arrayed on both sides of the question; the reasons on which he grounds his opinion of their malignant character are these:

"1. They possess the structure of compound adventitious cysts, the basis of this class of heterologue formations. 2. In the colour of the contained mass, and in the arrangements of the membranous septa or bands, the containing tissue; they are identical with scirrhus. 3. In hardness, occasionally justifying the application to them of the term stone cancer: they are not to be distinguished from the varieties of carcinoma already mentioned. 4. They occur very frequently in conjunction with growths of undoubted malignancy in other parts of the uterus. 5. And lastly, they possess an especial attribute of malignancy, incurability." (p. 289.)

He might have quoted the statement of Professor Valentin* in support of the opinion of the malignancy of these tumours, whose investigations have led him to conclude that their structure coincides in every respect with that of true scirrhus.

These growths are attended by an entirely different class of symptoms,

* Müller's Archiv, 1838, patholog. Jahresbericht, p. 59; and Valentin's Repertorium, 1837, p. 274.

according as their development takes place towards the surface of the uterus, or towards its cavity, constituting in the one case *subperitoneal*, in the other *submucous* tumours. The early stages of both forms are often unattended by any symptoms, and even when they have attained a very considerable size, they frequently occasion little inconvenience, except what results from mechanical pressure. The occurrence of pregnancy, however, in a uterus which is the seat of one of these tumours must be regarded as a great calamity. The increased supply of blood which the uterus then receives gives rise to inflammation and softening of the tumours, and if they be of large size, to death soon after parturition. This circumstance suggested to Dr. Ashwell the propriety of inducing premature labour in cases where these tumours are known to exist, in the hope of thus avoiding that disintegration of their texture which seldom fails to occur in the latter months of pregnancy, and the consequent danger to life which ensues if parturition takes place at the full period. He published his views on this subject some years ago in the 'Medical Gazette,' and their propriety is now generally acquiesced in by the profession. The justification of this practice rests mainly on the following facts: that death after labour complicated with these tumours is almost entirely attributable to the softening and unhealthy suppuration that go on in the growths themselves, the uterus being but little affected. And further, that these changes do not commence early in pregnancy, but that utero-gestation may often proceed as far as the sixth or seventh month without occasioning serious injury, while premature labour then induced rarely gives rise to constitutional mischief, may sometimes save the child, and will generally preserve the mother. In estimating the peculiar danger of labour thus complicated when it occurs at the full time, it should be borne in mind that whereas in difficult labour from pelvic distortion the uterine action is generally efficient, and the amount of the difficulty is appreciable during the progress of the labour, so that the forceps or perforator can be employed according as the one or other may seem to be indicated; the reverse of all this is the case where uterine tumours are present. The action of the womb is generally feeble, as though the fibres of the organ were in a measure paralysed by the morbid growths; and the degree of mechanical impediment cannot be correctly estimated, nor the extent of injury that may be inflicted on the tumours foreseen. Under such circumstances Dr. Ashwell advises the induction of premature labour, without reference to the fœtus, in order to preserve the mother's life. This recommendation accords with the principles of British midwifery, and is too in our opinion much more agreeable to the dictates of sound morals and humanity, than that sentimentality which in France has so long opposed the practice, but which is disappearing even there, under the common sense of Professor Dubois. The operation, however, is not to be resorted to indifferently wherever pregnancy coexists with a tumour of the uterus or its appendages. "If the tumour be moveable; if it be so situated that the enlarging and hard uterus has pushed it out of the way, and does not make painful pressure upon it; if it be the first labour so complicated; or if it be the second or third, the growth not having increased during the intervals of the successive pregnancies, then 'sequere naturam' should be the guiding precept. If, however, the opposite of these circumstances present themselves, it would be unwise to run the risks which a labour to be naturally prosecuted at the full time could not fail to induce." (p. 330.)

Nor is it a question of less importance to determine the period of pregnancy at which it may be expedient in each case to bring on premature labour. Something must depend on the time when the practitioner first sees his patient. If she have come early under his notice, and if he possess the tact requisite for making an accurate examination of the tumour and its attachments; and if he have accurately noted the first attacks of pain in it, and the constitutional impression produced by them; he will not hesitate much as to the precise period when to adopt this measure. "I should not delay, if there were constant pain in or near the growth; if the respiration were embarrassed; and if, as a consequence of these conditions, the pulse were quick and irritable, the extremities œdematous, and the functions of the kidney and skin were partially or greatly interrupted. When such an amount of evil exists, or rather before the entire of these symptoms is complete, the moment has arrived to empty the uterus; and I venture this opinion with the more confidence, conceiving that pregnancy will rarely give rise to this amount of mischief till the sixth or perhaps the seventh month of gestation; when if turning shall be required it may be accomplished with only the usual difficulties and hazards." (p. 333.)

In the treatment of these tumours when unassociated with pregnancy, Dr. Ashwell has obtained very encouraging results by the use of iodine.* He employs it both externally and internally, and has found it, when judiciously administered, very seldom produce constitutional injury. He has known tumours of the cervix uteri entirely disappear under its use, and when seated in the walls, or projecting into the cavity of the uterus, has found their further growth to be controlled by the same remedy; though, when occupying the latter situations, they do not become resolved or disappear. One case which he relates would seem to render it probable that the action of iodine upon these growths is sometimes very energetic, though the history of a patient suffering from this disease, communicated by Sir J. Clark to Dr. Ashwell, proves that the spontaneous breaking down of the structure of the tumour may take place wholly independent of the influence of any medicinal agents. Dr. Ashwell's case is that of a young unmarried woman, who, during eight months, took iodine as a remedy for a large tumour supposed to proceed from the uterus. She had, during this time, been an in-patient of Guy's Hospital, and on leaving that institution, she found the tumour so much softer as well as diminished in size, that she imprudently increased the dose of the tincture, and began to rub in very large quantities of the ointment. She had not long continued this dangerous practice, before she was attacked by all the symptoms of poisoning with iodine, and while suffering from them, she was again seen by Dr. Ashwell. Uterine hemorrhage had come on while she was taking the medicine, and on inspecting the discharge, it was found to contain masses of various sizes of broken down scirrhous growth. These continued to be discharged for

* An Essay on these tumours, by Dr. W. C. Roberts, in the New York Journal of Medicine for Sept. 1844, contains further proof of the good effects of iodine in their treatment.

some time; and when Dr. Ashwell ceased his attendance some months afterwards, no perceptible enlargement of the abdomen existed.

The submucous variety of these tumours is less within the control of remedial measures than the subperitoneal. Though their symptoms are generally not alarming, they sometimes give rise to very serious hemorrhages, which have been known, by their frequent occurrence, to prove fatal. They differ in structure from ordinary polypi of the uterus, nor do they, except in very rare cases, become pediculated. They differ, too, in sensibility and in vascularity; the alarming hemorrhages which they sometimes occasion, coming entirely from their investing membrane, while their substance appears to be wholly destitute of vessels. In many instances, their connexion with the uterus is not such as to admit of their removal by ligature; and even when its application is practicable, far less favorable anticipations must be formed of the result than when it is used to remove an ordinary polypus. In most instances, palliative treatment to restrain their growth and check the hemorrhage to which they may give rise is all that can be attempted.

From organic diseases of the uterine parietes, Dr. Ashwell passes to those affecting the os and cervix uteri, and enters on the consideration of cancer of the womb. He asserts the curability of this disease in its early stage, and supports this opinion partly by analogical reasoning, founded on the results obtained by the use of iodine in the treatment of hard tumours of the uterus, partly by the statements of Montgomery and Duparcque. There is a vagueness, however, in the manner in which M. Duparcque speaks of "the greater part of confirmed cancers of the womb, succeeding to congestion and ulceration, capable of being cured," which is very unsatisfactory. Neither does Dr. Montgomery's account of what he conceives to be the first stage of cancer of the womb appear to us by any means conclusive. He is of opinion* that the disease begins in the glands of Naboth, which may be felt hard, like small shot, owing to thickening of their coats by the deposit of scirrhous matter. It is unnecessary to trace his description of the various stages through which, from this commencement, he supposes the disease to pass, for this theory is too much at variance with what we know of the mode in which cancer commences in other tissues for us to subscribe to it without more conclusive evidence than is afforded by the two illustrative cases related by Dr. Montgomery. Whatever difference of opinion may exist as to the characteristics of the early and possibly curable stage of cancer, a sad uniformity prevails with reference to the signs that betoken all hope of cure to be vain, and all remedies inefficient. When once softening and abrasion have occurred, the palliation of symptoms becomes the sole care of the medical attendant. Still the opinion that a case is hopeless is not to be hastily formed, still less to be hastily expressed.

"It would be as wrong even in such a state, at once to announce its real character, as it would be to abandon the patient to insufficient and partial efforts of palliation. There is yet much to be done; and as it is impossible to say how long life may be protracted, so we ought by every possible means, to smooth the approach of death. Lately, in a case where the hemorrhages although small were frequent, and where the os was fissured and granulated, ulceration being erroneously supposed to exist, an abandonment of all active treatment was recom-

* Dublin Journal of Medical Science, Jan. 1842.

mended. And yet in this instance, the application of a very strong solution of lunar caustic, daily injections of a saturated alum lotion, a regulated diet, and a sparing exhibition of narcotics have kept the disease stationary for three years,—four months having been predicted as the utmost extent to which life could possibly be protracted. Such opinions should not be hastily given. There is neither empiricism nor fraud in an opposite course. It is certainly wrong to promise a cure; but if life can be prolonged for months without painful treatment, and with some considerable measure of comfort, it is right to afford the patient such a chance of an arrest of the malady." (p. 406.)

The management of the discharges in cases of ulcerated carcinoma is a matter of great practical moment, and one on which the opinions of a man of large experience are well worth learning. Dr. Ashwell justly censures the indiscriminate employment of stimulating and astringent injections, and recommends that so long as the discharge is not excessive, acrid or offensive, tepid or cold water be used once or twice a day. This simple measure often procures to the patient much comfort; but when the discharge becomes excessive or offensive there is generally an anxiety to employ injections. When the discharge is thin and ichorous, and the parts relaxed, Dr. Ashwell has found the following injection used once daily, or two or three times a week, very serviceable.

℞ Sinapis pulver. ʒij.

Aquæ ferventis, ℥xvj. M. ft. injectio.

If reduced to such a strength as only to excite a little tingling, he has seen this remedy if used at the very commencement of the ulceration arrest and improve the character of the discharge and allay pain. As narcotics, to be used at a temperature regulated by the patient's feelings, and the amount of hemorrhage, he employs the following.

℞ Liq. Opii sedativ. ♏ xxx.

Inf. Valerianæ, ℥j.

Mucil. Acaciæ, ℥ss. M. ft. Enema.

℞ Flor. Anthemidis.
Semin. Lini Cortus. aa ℥ss.
Aquæ fervid. ℥vj. Macera et cola, dein adde Opii, gr. ij, iij, vel vj. Half to be used at a time.

℞ Fol. Belladonnæ Exsicc. gr. xij.

Aquæ Fervid. ℥vj. M. ft. Enema.

or ℞ Olei Terebinth. ℥ss.
Ovi unius Vitellum. Tere simul et gradatim adde Decocti Hordei tepid. ℥x.

His favorite astringents, especially in the earlier periods of the confirmed malady are

℞ Decoct. Secalis Cornut. ℥xiv.

Argenti Nitrat. gr. xx.

Tinct. Catechu, ℥ij. M. ft. Injectio vaginalis.

Four ounces to be used three times a day. The decoction is prepared by boiling an ounce of the bruised rye in a pint and a half of water, down to a pint.

℞ Inf. Quercus, ℥iv. Pulv. Gallarum, gr. xxx.

Tinct. Catechu, ʒij. M. ft. Injectio vaginalis.

℞ Argent. Nitrat. gr. xv. ad ʒj.

Aquæ Rosæ, ℥xvj. M. ft. Injectio vaginalis.

Three or four ounces to be used three or four times daily.

In the employment of injections, however, it should be borne in mind that if they fail to check the amount and diminish the fætor of the discharge and especially if prolonged pain succeed their administration, they are doing harm and should be discontinued. Dr. Ashwell appears like Dr. Churchill to have seen much relief follow the use of strong solutions of the nitrate of silver, both in diminishing the quantity, odour, and acrimony of the discharge, and has observed results equally favorable from the use of a solution of ʒj, or ʒiss of the sulphate of iron to a pint of distilled water. With the advance of the disease, however, the use of injections must be laid aside in consequence of the pain and bleeding which follow their administration. Tepid water, or other unstimulating applications must, however, still be used as ablutions.

We have condensed Dr. Ashwell's remarks on this subject as much for the illustration they afford of the practical tendency that pervades the whole book as for their own intrinsic value. There are many more observations on the management of cancer uteri which we could have wished to extract; but to which we must content ourselves with referring.

From carcinoma the author passes to simple ulceration of the os and cervix, which he treats of briefly, and then taking up the consideration of corroding ulcer of the uterus, reverts to malignant diseases of the organ. We cannot but think that without at all diminishing the practical value of the work, it would be possible to make some nearer approach to a scientific arrangement of its contents than Dr. Ashwell has attempted. Both corroding ulcers and cauliflower excrescence of the uterus are extremely rare diseases, especially the former, of which Dr. Ashwell has seen but two examples in twenty years. Both these affections are regarded by him as of malignant character. He states that in those cases in which he has examined malignant ulceration of the cervix uteri by means of the speculum, he has been struck by its close resemblance to lupus in external parts; to which the whole character of the morbid processes in this disease seems very analogous. His remarks on these affections contain nothing very novel; he considers both, however, to be more amenable to treatment in an early stage than other forms of malignant disease.

The succeeding chapter on *occlusion and rigidity* of the os uteri is one of great practical importance. Dr. Ashwell shows that occlusion of the os uteri is by no means so hypothetical an occurrence as has been imagined by some writers; and criticises, in our opinion with great propriety, the supposition that most of the alleged cases of imperforate os have in reality been instances in which the orifice of the uterus was removed from reach by extraordinary obliquity of the uterus. It is our belief indeed that obliquity of the uterus has had an importance attached to it in books far greater than was ever substantiated by observation of nature's proceedings. The diagnosis, then, being attended with no great difficulty, the treatment to be adopted becomes the next question, and is one on which there exists considerable difference of opinion. Some authors, among whom is Naegele, recommend the employment of pressure by the finger, a female catheter, sound, or bougie; while others with Dr. Ashwell advocate the use of the knife. The author shows that Naegele's mode of proceeding is applicable only to those cases in which the occlusion is very slight; since the forcible use of the finger or of a catheter to make an artificial os must cause great contusion, and render the supervention of uterine inflammation extremely probable. But, not merely does he consider the use of the knife to be indicated in cases of actual occlusion of the os, he regards its use as justifiable in that far more frequent class of cases in which the orifice of the uterus is excessively rigid and undilatable. In such cases three alternatives present themselves; either incisions may be made into the os uteri, or artificial dilatation may be attempted, or the patient may be left to nature, which indeed amounts to little else than abandoning her to certain death. Forcible dilatation of the os uteri in such a case has happily now become matter of history or nearly so; but a degree of apprehension exists with reference to the possible hazards of incision, which may lead to too great reliance on other means, and to too tardy a recourse to the operation. On this point Dr. Ashwell gives the following useful cautions:

"It may then be assumed, that we are not justified in protracting the employment of the knife till the patient is nearly exhausted by the continuance and severity of the expulsatory efforts; the indications of approaching collapse being apparent in a quick and feeble pulse, a cooling surface, hurried and short respiration, a subdued tone of voice, a tender and tympanitic abdomen, and a gradually diminishing uterine pain. Many instances are on record precisely of this kind; and the event, in nearly all proved fatal. Nor ought we to hesitate about incising the cervix, when the violence and frequent return of the uterine effort threatens rupture of the womb. If there be distressing and constant pain about the neck or body of the uterus, or in any other part; if the countenance become turgid and dark; if perspiration issue at every pore, and the pulse be full, strong, quick, and incompressible; and if these symptoms continue, although perhaps somewhat lessened by bleeding and antimony, there can be no doubt that recourse should be had to incision. It is impossible to fix a precise limit during which a patient may be safely left to unaided efforts: time is not the only condition, although an essential one in every rule regulating interference in obstetric cases.

"There can be no doubt that in many instances of rigidity, a free abstraction of blood, the exhibition of one sixth, one fourth, or one half grain every hour of tartarized antimony, with or without opium, till it produce nausea, will accomplish the dilatation. No sensible practitioner would feel himself warranted at once to propose incision; nor would such an individual consider himself justified in not performing it when other means had failed. While, on the one hand, I am anxious to avoid the imputation of rashness, I am, on the other, if possible, more desirous to avoid the imputation of timidly shrinking from a procedure absolutely essential to a patient's welfare." (p. 453-4.)

Dr. Ashwell next describes the mode of performing the operation, but this is so simple a proceeding that we need not enter into detail respecting it. It may, however, be well to mention that the extension of the incision by laceration very rarely reaches beyond the limits of the vagina, and that much apprehension need not, therefore, be entertained on that score.

We now come to the third part of the work, which has but just appeared. It opens with a chapter on *polypus uteri*, in which we do not find anything of particular importance. For the removal of these growths, Dr. Ashwell prefers the ligature to excision, and in tying them he employs Gooch's double canula, with a little windlass appended to it, for the sake of greater convenience in tightening the thread.

*Physometra* and *hydrometra* are the subjects of the next chapters.

Dr. Ashwell has never met with an instance of idiopathic uterine tympanites, but does not, on that account, deny its possibility, as has been recently done by Profs. Naegele and Stolz. Authentic cases of physometra are on record; the most recent one with which we are acquainted was related by M. Tessier, in the 'Gaz. Médicale de Paris,' for January 1844; and on account of the rarity of the affection, it may be worth while to extract the patient's history. She was a married woman, aged 43, and came under M. Tessier's care for chronic metritis associated with hysterical symptoms. At that time there existed considerable and easily appreciable swelling of the body and cervix of the uterus; the patient had suffered from leucorrhea for fifteen years, and had not menstruated for two and a half months, and she complained of pain in the sacrum and hypogastric region, and had frequent attacks of hysteria.

In the course of a few months, the abdomen began to swell, and the leucorrhea diminished considerably. The size of the abdomen now began to increase rapidly, and as the menses did not reappear, the patient suspected that she was pregnant. Examination per vaginam ascertained that the uterus was considerably enlarged; but it was easily raised by the finger, and percussion in the hypogastric region yielded an uniformly clear sound.

The menses had been absent for six months; the uterus had reached nearly as high as the navel; the patient believed herself to be undoubtedly pregnant, and stated that she had experienced sensations similar to those produced by the movements of a child. M. Tessier, however, thought her to be suffering from tympanites uterina, for neither the placental souffle nor the pulsations of the fetal heart were perceptible on auscultation; the abdomen yielded everywhere a clear sound, and the uterine tumour was remarkably light. At this time the patient was seized one evening with violent pains like those of labour, proceeding from the sacrum and umbilicus, and shooting into the pelvis, which were immediately followed by the expulsion of a large quantity of offensive air from the vagina. During the escape of this gas, the abdominal tumour diminished in size, and in the course of a few hours had totally disappeared. The escape of this gas was not attended with the discharge of coagula, or of a mole, or of any other foreign body to the putrefaction of which its generation might have been attributed. Three years had passed from this occurrence to the date of M. Tessier's report, in which he mentions that his patient has not become pregnant, but that the above-mentioned phenomena had occasionally recurred.

Dr. Ashwell relates an equally indubitable case of *hydrometra.*

"Miss —— is 29 years of age, stout, and has hitherto enjoyed tolerable health; menstruation has often been irregular, although generally natural in amount and character. Five months ago she first discovered that during the flow of the catamenia there were discharges of water, which ceased when the period was over. Soon however, they appeared in the interval, and for the last eight or ten weeks she has never been free from watery discharges, except during the night. On no occasion has it been tinged with blood, and a careful examination shows that the secretion is limpid, very thin, entirely colourless, and free from odour. Nine or ten napkins, sometimes more, are soaked through in the twenty-four hours. Her aspect is unhealthy, her colour gone, and she is losing flesh; the pulse is quicker than natural, but the appetite has not much failed, nor are the bowels constipated; the urine is scanty, not high coloured, and there is increasing weakness.

"The cervix uteri, indeed the whole organ as well as the vagina, is healthy, not enlarged, nor at all tender; but having the feel of parts constantly under discharge. Alum and catechu are taken internally, and the strong alum hip-bath is used every day, with a generous diet. These means improved the health, and as, during the night, the discharge ceased, the patient was directed to keep her bed, and steadily to persevere with the remedies. At the expiration of a fortnight the catamenia returned, and during the four or five days they continued to flow, there was watery discharge only for two hours. The measures formerly adopted were then resumed, and there was no return of the serous secretion. When I last heard of this young lady, she had discontinued the use of medicine, and was attending to her usual duties." (p. 508-14.)

A condition of more frequent occurrence than hydrometra of the unimpregnated womb, and consequently of greater practical moment is one first described by Dr. Ashwell in the Medical Gazette, under the title of aqueous discharge from the uterus after parturition. It consists in the discharge soon after parturition of large quantities of aqueous fluid, which come away at first by gush, and afterwards continue to drain for a considerable time, the true lochial discharge and the secretion of milk being at the same time suppressed. The other symptoms attending it, seem to indicate its inflammatory origin, and a modified antiphlogistic plan has been the mode of treatment which has seemed best adapted to remove it. Dr. Ashwell himself has not met with any fatal case of this affection, but he details the history of a patient communicated to him by Mr. Bury of Farnham, in which death from exhaustion having followed the symptoms, three masses of a fungoid growth were found springing from the lining membrane of the uterus.

When treating of *prolapsus uteri*, Dr. Ashwell defends the use of pessaries with great good sense from the attacks of the late Dr. Hamilton. We have, however, already extended our notice to such a length that we can merely direct the attention of those among our readers to it, whose faith in their utility may have been shaken by the objections of the late talented but somewhat eccentric professor of midwifery at Edinburgh.

*Hypertrophy of the cervix uteri*, so readily mistaken on a superficial examination for prolapsus of the uterus, is treated of somewhat too cursorily. Dr. Ashwell does not appear to be acquainted with the very valuable paper of Dr. Evory Kennedy on this subject.

In treating of displacements of the uterus, *inversion* of the organ comes next under consideration. Dr. Ashwell coincides in the opinion that its spontaneous occurrence is by no means infrequent, and in proof of this relates two striking instances which came under his own notice. He shows that the rapid dissolution which sometimes succeeds inversion of the uterus is by no means invariably attributable to the amount of hemorrhage which takes place; but is rather owing to the violent shock communicated to the whole nervous system by the accident. Hence the vast importance of the administration of stimulants, and of the immediate reduction of the displaced organ. In connexion with this subject there is one point on which much discrepancy of opinion has existed, and still prevails; namely whether the placenta should or should not be detached from its adherence to the womb, previously to any attempts at reduction. On the one hand, the danger of immediately fatal hemorrhage has been urged as an objection to its detachment; on the other, the difficulty, sometimes indeed the impossibility, of returning the uterus while its bulk is increased by the placenta is assigned as a reason for its separation. The question appears to us to be very fairly stated, and wisely answered in the following paragraph.

"Whether the placenta, if attached, should be separated prior to reduction, is a question on which there has been considerable difference of opinion. In neither of my cases, although I tried for some minutes, could I return the uterus with the placenta; but in neither did hemorrhage precede or follow its separation. Where the os is firmly contracted, there is little risk of bleeding, for the uterine vessels are effectually constricted at the upper part of the tumour. Reduction must be facilitated by the previous removal of the placenta, adhering as it generally does, to the fundus, the part to be first returned. Denman cautiously remarks, 'that where the placenta is partly separated, it would be proper to finish the separation before reduction is attempted; but if the placenta should wholly adhere, it will be better to replace the uterus before we endeavour to separate the placenta.' This is not sound advice; for when the placenta is thus partially separated, hemorrhage is either present or highly probable. Now if there has already been bleeding from the partial detachment of the viscus, and we know how rapid floodings sometimes are, it certainly would be very unsafe, by completing the detachment of the placenta, to uncover more of the uterine sinuses, and thus to increase the patient's danger. The better practice would be at once to attempt the reposition, trusting to uterine contraction being excited, so soon as the organ was returned into the pelvic cavity, this great point being additionally secured by the stimulus of the hand within. Denman urges as the ground of his advice 'that while we are separating the placenta, the cervix of the uterus is speedily contracting, and the difficulty of replacing it increasing, which is a far greater evil than a retained placenta.' When the cervix thus contracts, and goes on contracting, the risk of hemorrhage is slight, and the difficulty of reduction great; a difficulty which should not be increased by permitting the placenta to augment the size of the already too bulky uterus.

"We may then conclude, that where the protruded organ and its attached placenta do not together make up a large tumour, and where the os does not constrict its upper part, reduction of the whole may be at once attempted; where, however, these favorable conditions do not exist it will be wiser to insure reposition by previous separation." (p. 579-80.)

We pass over a very curious case of anteflexion of the uterus communicated by Dr. Walshe to Dr. Ashwell, in order to notice his remarks on the management of retroverted uterus. A notion has become somewhat too general, that it is sufficient in most cases of retroverted uterus to keep the bladder duly emptied by means of the catheter; and that by attention to this point the uterus will sooner or later return to its natural position. Now it is beyond all question that spontaneous reduction of the uterus has in several instances followed the repeated use of the catheter. But while the importance of attending to the state of the bladder cannot be over estimated, Dr. Ashwell remarks with great justice that the complete emptying of the bladder by means of the catheter is almost impossible; and that the bladder itself is exposed to serious injury from the gradual accumulation of urine within it, that must consequently take place. Nor indeed is this all, but it is by no means certain that the patient's sufferings will subside, or that the uterus as it becomes developed will return of its own accord to its proper situation, and no more distressing case can be conceived than one in which retroversion persists to the end of pregnancy. Compared with this danger, the risk of producing abortion by interference, and the occasional difficulty of replacing the uterus are but slight evils.

"In 1840," says Dr. Ashwell, "I was requested to see a young woman four months pregnant. Nearly a fortnight before, soon after jumping over a flower-pot in the garden, she found she could not pass her urine. She strained violently, sat over hot water, and applied gin fomentations, but all without effect. A neighbouring practitioner discovered the retroversion, drew off the water, gave an aperient, but did not attempt reposition. The symptoms grew daily much worse; the use of the catheter was attended with great suffering, and was always followed by the passage of purulent matter, and frequently of blood. Defecation could only be effected by active purgatives, for it was impossible to inject a clyster. The pulse was quick and compressible, there was constant nausea and frequent vomiting. What was to be done? I urged immediate reduction; but the attendant practitioner recommended still further delay, expressing a wish that after the use of the catheter, she should lie on her abdomen to aid spontaneous replacement. To this, most unwillingly, I was compelled to assent. Two days elapsed, bringing with them not the reduction, but aggravated sufferings. My first attempt was unsuccessful; on the next occasion, with as little severe pressure as possible, I got nearly the whole of my right hand into the rectum, while with the fingers of my left hand I tried to reach and press upon the cervix. In less than a quarter of an hour, or very little more, I succeeded. The patient, although complaining of the process, declared the suffering was nothing in comparison with what she had endured the previous three weeks. There was no amelioration to be hoped from palliative measures; every day would have accumulated difficulty and suffering; and at length, long before the term of pregnancy had expired, either puncturing the membranes or tapping the uterus, must have been resorted to." (p. 607, note.)

This case may be taken as an illustration, we think, too, as a justification of Dr. Ashwell's practice; and we believe that his estimate of the evils likely to follow from the continuance of retroversion are by no means exaggerated; while in those cases, where this accident occurs after the fourth month of pregnancy, the womb may become so impacted in the pelvis that nothing short of the introduction of the hand into the rectum will suffice to accomplish its reduction. Of course this manual interference would not be attempted without the previous use of bleeding, the warm bath, or antimony, means which greatly tend to facilitate reposition. For cases and arguments in further justification of this practice we must refer our readers to the book itself; and hasten in conclusion to notice his remarks on *ovarian dropsy*. We are glad to find Dr. Ashwell advocating the same opinions with reference to the operation of extirpation as we expressed in this Journal in Oct. 1843, and Jan. 1844. The very objections that we then stated to the proceeding are assigned now by one whose experience in the management of female diseases surpasses that which falls to the lot of most; nor is it a point of small moment to bear in mind that as the cases of this operation have become more numerous, the average of fatal cases has likewise risen; a proof beyond dispute that the opposition we then expressed to the proceeding had a basis far more solid than mere prejudice.

"These considerations," says the author, after mentioning cases in which the disease has remained stationary for years, "are entitled to great weight when determining the propriety of extirpation, uncalled for by present and great evils; or where the operation from the enthusiastic views of its patrons is urgently recommended as a preventive of mischiefs which they deem, but not always on good grounds, to be prospectively inevitable. To operate, where the patient strongly desires it, from a conviction that her sufferings and the frequent repetitions of paracentesis, will otherwise prove speedily fatal, may not involve any distressing responsibility, especially where the condition of the tumour leads to the supposition, that the case is pathologically a favorable one. But there are examples selected for operation far different from this. Take, for instance a case which occurred to me a few months ago. A lady travelled to town from a considerable distance, anxious to have extirpation performed. On inquiry, I found she was 62 years of age, had never been tapped, although ovarian dropsy had existed for more than half her life. There was scarcely any suffering beyond weight and pressure, although the tumour was of immense size and partly solid. In such a case it would have been highly culpable to have operated; and yet a surgeon, over zealous about the removal of ovaries, had induced the firm belief that it ought to be done. I need scarcely add that the patient after being made acquainted with the great danger of the operation was perfectly satisfied to remain as she was. Nor will the practitioner be less perplexed and distressed by such a case as the following, which occurred within my observation not long since—a young woman, under 22 had ovarian dropsy; her countenance bespeaking excellent health, and her history confirming the impression. Without interference, many years might have been added to her existence; and as one of the fortunate incidents of life, it might have so happened that the tumour should cease to grow. But unhappily she was convinced that extirpation was proper; the operation was most ably performed, and in a few days she died. These certainly are not the cases in which removal ought to be practised. If the operation is to become established, of which I have the strongest doubt, it must be confined to examples of the malady where tapping has been already so often performed as to preclude, from the experience of similar cases, any idea that it can ever be dispensed with; and where, we are confident, that great suffering must lead to early death. Perhaps this may be regarded as too limited a view of the value of extirpation, but it is, I think, the correct one. In such cases, if the diagnosis excludes the belief that there are serious adhesions, or malignant or solid growths complicating the tumour, and if the patient strongly desires it, the operation is defensible. In all other examples it can only rest on the patient's own views of her future prospects, and on a calculation of chances. She might live several years and without much suffering; but she may die in a few years after great suffering; she determines, therefore, being courageous, and probably strongly urged by her surgeon, to run the risk of immediate death for the hope of immediate and radical cure. Whether she has done wisely to submit to such a hazard a successful operation can scarcely prove; that she has happily secured her safety, through imminent peril, such an operation does prove. Lithotomy, operations for hernia, and for securing large arteries, rest on different grounds. That they are essential to the patient's life, is a full justification of their performance; for in all, even if not dangerous at the moment, it is certainly known that life will soon be destroyed, either by fever, gangrene, or loss of blood. Such, it has been proved, has not been the case in many of the fatal operations lately performed for extermination of ovarian encysted tumours. It does not appear that statistics more favorable even than we have any right to expect, will materially change the aspect of the circumstances under which the operation is to be performed. It most probably will, from the impossibility of determining the real character and adhesions of the growth, ever remain an eminently uncertain operation. The extirpation, we are assured, by the operators themselves, in a fit case, is far from difficult: would that it were more so, for then it would not readily be undertaken! If it required as much surgical knowledge and skill to make these large and brilliant abdominal incisions as to tie the subclavian artery or to perform a trying operation of lithotomy, the lives of many women would have been already spared, and fewer would be sacrificed for the future. What would be thought of the feasibility of any other operation involving life in the most imminent hazard, if we discovered that out of sixty-seven cases where it had been attempted, it was *from absolute error of diagnosis, incapable of completion in eighteen;* that of the remaining forty-nine patients, where the extirpation was effected, *sixteen died,* and *two were not cured;* so that out of the whole number, sixty-seven, *the operation failed in thirty-six,*

and *succeeded in thirty-one*, less than one half. Such results are distressing, especially when we hear no greater doubt expressed about the operation itself, but only higher confidence in its value, and greater laudation of the operators. We willingly concede presence of mind and ability to many of the extirpators of ovarian cysts; but we are unable to discover (for the later operations have been quite as unsuccessful from unfitness of the cases as the earlier ones,) that any advance has been made in diagnosis. Nor when the tumours themselves are examined after death, when the malignancy of many of them is recognized, and their firm, almost indivisible adhesions, and their immovable masses of new and morbid substance are brought to view; it is next to impossible to entertain any sanguine hope, that our means of diagnosis can ever be much improved. (pp. 647-9.)

A brief notice of the more important diseases of the external organs occupies the last thirty pages of the book. Into them, however, we have not space to enter, but must here part company with Dr. Ashwell, with the hope that he will not delay in giving to the profession the results of his experience in the diseases of the pregnant and puerperal states.

---

